# Computed tomography features and clinicopathological characteristics of resectable esophageal sarcomatoid carcinoma: a retrospective study

**DOI:** 10.3389/fonc.2025.1655957

**Published:** 2025-12-05

**Authors:** Meng Li, Yang Li, Mingbo Wang, Huiyan Deng, Xiangming Wang, Gaofeng Shi, Andu Zhang, Jialiang Ren

**Affiliations:** 1 Department of Computed Tomography and Magnetic Resonance Imaging, The Fourth Hospital of Hebei Medical University, Shijiazhuang, Hebei, China; 2 Department of Thoracic Surgery, The Fourth Hospital of Hebei Medical University, Shijiazhuang, Hebei, China; 3 Department of Pathology, The Fourth Hospital of Hebei Medical University, Shijiazhuang, Hebei, China; 4 Department of Radiotherapy, The Fourth Hospital of Hebei Medical University, Shijiazhuang, Hebei, China; 5 Department of Pharmaceuticals Diagnostics, GE Healthcare China, Beijing, China

**Keywords:** computed tomography, clinicopathological, resectable, esophageal, sarcomatoid carcinoma

## Abstract

**Objectives:**

Esophageal sarcomatoid carcinoma (ESC) is a rare malignant tumor. This study aims to analyze the computed tomography (CT) features and clinicopathological characteristics of resectable ESC.

**Methods:**

The CT and clinicopathological data of 25 patients with ESC, confirmed by postoperative pathology, were retrospectively analyzed. The preoperative CT feature analysis included the average tumor CT attenuation value (CT_Tumor_), the average normal esophagus CT attenuation value (CT_Norma_l), the tumor-to-normal esophagus attenuation ratio (TNR), enhancement pattern, tumor margin, and tumor morphology, which was classified into two types: mass-forming type and wall-thickening type. Additionally, CT-measured tumor thickness (cTT), CT-measured tumor length (cTL), and CT-measured tumor volume (cTV) were measured and recorded. The analysis of clinicopathological characteristics encompassed variables such as age, gender, clinical symptoms, pathological tumor thickness (pTT), pathological tumor length (pTL), T stage, N stage, lymphovascular invasion status, and neural invasion status. To assess the agreement and correlation between CT features and pathological results, Bland-Altman plots and Pearson correlation coefficient analyses were performed for pTT versus cTT and pTL versus cTL, respectively.

**Results:**

Among the 25 patients with ESC, 19 were male and 6 were female. The patients’ ages ranged from 47 to 73 years, with a mean age of 65.48 ± 6.85 years and a median age of 68 years. The pathological staging results showed that 14 cases were at stage T1, 5 cases at stage T2, 5 cases at stage T3, and 1 case at stage T4. Lymph node metastasis was identified in 12 cases, including 5 classified as N1, 4 as N2, and 3 as N3. The pTT ranged from 0.50 to 4.00 cm, with a mean of 1.92 ± 1.02 cm and a median of 1.50 cm; the cTT ranged from 0.60 to 4.10 cm, with a mean of 2.02 ± 0.90 cm and a median of 1.66 cm. Bland-Altman analysis demonstrated a mean difference of 0.10 cm between cTT and pTT, with 92.0% (23/25) of cases lying within the 95% limits of agreement (Mean ± 1.96 SD). The Pearson correlation coefficient between the two measurements was 0.980, indicating a strong positive correlation. The pTL ranged from 2.00 to 7.00 cm, with a mean of 4.24 ± 1.50 cm and a median of 4.00 cm; the cTL ranged from 2.57 to 7.50 cm, with a mean of 4.41 ± 1.48 cm and a median of 4.16 cm. Bland-Altman analysis demonstrated a mean difference of 0.18 cm between cTL and pTL, with 92.0% (23/25) of cases lying within the 95% limits of agreement. The Pearson correlation coefficient was 0.884. The cTV ranged from 1.56 cm³ to 41.49 cm³, with a median of 8.25 cm³. Regarding tumor morphology, 20 cases presented as mass-forming type, while 5 were classified as wall-thickening type. Significant differences in both pTT and cTT were observed between the two morphological types; however, no statistically significant differences were found in other CT features.

**Conclusion:**

ESC is a rare malignant tumor characterized by distinctive CT features. The majority of cases manifest as the mass-forming type, while a smaller proportion present as the wall-thickening type. The definitive diagnosis of ESC depends on pathological examination.

## 1 Introduction

In 2022, there were 511,000 new cases of esophageal cancer (EC) and 445,000 deaths globally, representing 2.6% and 4.6% of all cancer incidence and mortality, respectively, with EC ranking 11th in incidence and 7th in mortality among all malignant tumors worldwide (1). China bears the heaviest burden of EC globally; statistical analyses indicate that over half of the new cases and deaths worldwide occur in China (2). The incidence of esophageal cancer ranks ninth among all cancers, following lung cancer and breast cancer, while its mortality rate ranks sixth (3, 4). Globally, despite the implementation of multimodal treatment approaches and perioperative care, EC remains a devastating disease, with an overall five-year survival rate ranging from 10% to 25% (5, 6).

The primary pathological types of EC include esophageal squamous cell carcinoma (ESCC) and esophageal adenocarcinoma (EAC) (1). Primary esophageal sarcomatoid carcinoma (ESC) is an rare entity, accounting for approximately 2% of all malignant esophageal tumors. It is frequently misdiagnosed as conventional EC or other histological subtypes (7). However, sarcomatoid carcinoma (SC) is a rare and diagnostically challenging tumor that can arise in various anatomical sites typically affected by carcinomas, with higher prevalence reported in the head and neck region, lungs, and urinary bladder (8). The biological behavior and prognosis of ESC differ from those of more common EC types, such as ESCC and EAC. ESC is characterized by a lower metastatic potential, higher rates of complete surgical resection, and relatively favorable prognosis. Recent study have demonstrated that the three-year survival rate for ESC is significantly higher than that for ESCC. However, the five-year survival rates between the two are comparable, which may be related to the current lack of effective treatment strategies (9). Clinically and radiologically, ESC often presents with features similar to those of other esophageal tumors, rendering pre-treatment diagnosis challenging. Nevertheless, accurate imaging analysis plays a critical role in facilitating diagnosis and provides essential information for guiding clinical management and postoperative surveillance (10, 11).

Early detection of EC is crucial for improving survival rates and reducing morbidity and mortality (12). There is a substantial body of literature analyzing the imaging characteristics of ESCC. Recent advancements in medical imaging have significantly transformed the detection and classification of EC, providing new levels of precision and accuracy. Recently, studies utilizing deep learning models have employed various medical imaging data for the early detection of ESCC, developing multiple diagnostic models to enhance the diagnostic and therapeutic framework for EC (12–14).

However, ESC is a rare malignant tumor of the esophagus, and there is currently a lack of analytical literature providing a systematic description of its CT features, which are essential for accurate diagnosis. Therefore, this study aims to improve the diagnostic accuracy of ESC through a detailed analysis of the CT features in 25 cases confirmed by postoperative pathological examination. Furthermore, the findings are summarized in the context of the relevant literature. To the best of our knowledge, this study represents the largest case series to date focusing on the CT characteristics of ESC.

## 2 Methods

### 2.1 Patient selection

**Figure 1:**
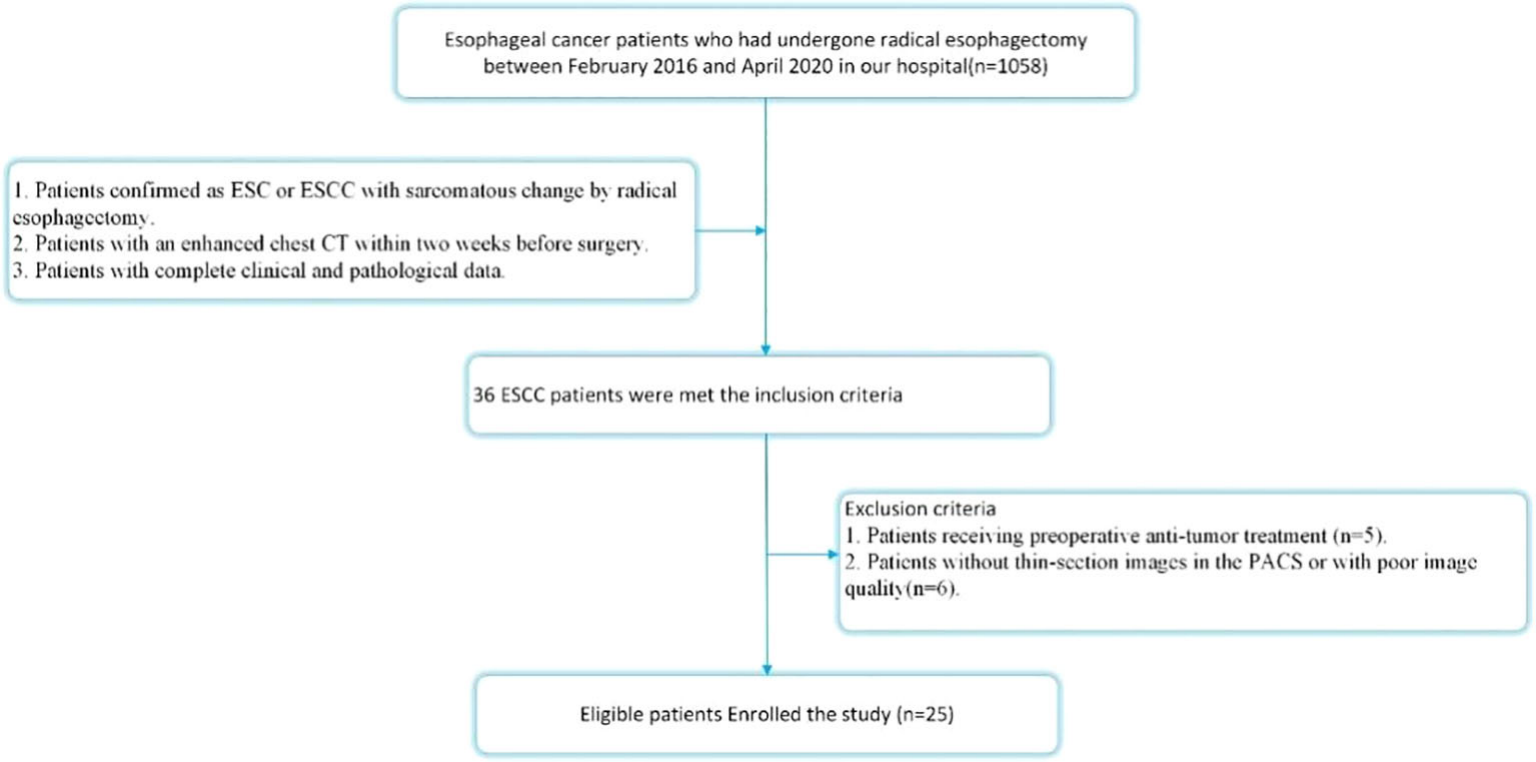
The flowchart of the patients enrolled in our study.

This retrospective study was approved by the institutional review board of our institution, which granteda waiver of informed consent. Between February 2016 and April 2020, pathology records and corresponding CT images stored in the Picture Archiving and Communication System (PACS) of our hospital were retrospectively reviewed using search terms including “esophagus”, “sarcomatoid carcinoma” or “ESCC with sarcomatoid change.” The inclusion criteria were as follows: 1. Patients pathologically confirmed as having ESC or ESCC with sarcomatous change following radical esophagectomy; 2. Patients who underwent chest contrast-enhanced CT (CECT) within two weeks prior to surgery; 3. Patients with complete clinical and pathological data. The exclusion criteria were: 1. Patients who received preoperative anti-tumor treatment (n = 5); 2. Patients lacking thin-section CECT images in the PACS or with poor image quality (n = 6). Ultimately, a total of 25 patients met the criteria and were included in this study. The patient selection process is illustrated in **Figure 1**.

### 2.2 CT images acquisition

All available thin-section mediastinal window chest CECT images were retrieved from the PACS of our hospital. Images were acquired using two commercially available CT scanners: a 128-slice second-generation dual-source CT scanner (SOMATOM Definition Flash, Siemens Healthcare, Germany) and a 256-slice CT scanner (Revolution CT, GE Healthcare, USA). Patients were positioned in a supine manner with the head advanced. The scanning range extended from the thoracic inlet to the level of the abdominal aortic opening, encompassing the entire esophagus. Imaging parameters were set as follows: tube voltage of 120 kV; tube current of automatic mAs; field of view of 500 mm; matrix size of 512 × 512; and section thickness of 1-1.5 mm. A dual-barrel automatic high-pressure injector was employed to administer the contrast agent (Iohexol, 350 mg/mL) via an antecubital vein at a dose of 1.2-1.5 mL/kg, with an injection rate of 3.0-4.0 mL/s. Contrast-enhanced scans were initiated with a 30-second delay, followed by a 20 mL saline flush at a rate of 3 mL/s.

### 2.3 CT features analysis

Two experienced radiologists, with 13 and 11 years of expertise in CT imaging of EC, independently conducted a retrospective analysis of the CECT images. All assessments were performed using RadiAnt software, with both radiologists blind to the patients’ clinical and pathological data. The quantitative analysis of CT features included the following parameters: average tumor CT attenuation value (CT_Tumor_), average normal esophagus CT attenuation value (CT_Normal_), tumor-to-normal esophagus attenuation ratio (TNR = CT_Tumor_/CT_Normal_), the difference between tumor and normal esophagus attenuation (ΔTN=CT_Tumor_-CT_Normal_), CT-measured tumor thickness (cTT), CT-measured tumor length (cTL), and CT-measured tumor volume (cTV). Both CT_Tumor_ and CT_Normal_ values were reported in Hounsfield units (HU). The cTT was measured on the axial plane exhibiting the largest tumor dimension, while cTL was measured on the sagittal plane demonstrating the greatest tumor length. The cTV was derived by manually outlining the tumor boundary on each transverse section and summing the volumes across the slices. Qualitative features included tumor necrosis, margin pattern (categorized as clear or unclear), and enhancement pattern (classified as homogeneous or heterogeneous).

Interobserver agreement for continuous variables was assessed using the intraclass correlation coefficient (ICC). An ICC value greater than 0.75 was considered indicative of good reliability, and in these instances, the mean of the two measurements was used for subsequent analyses. For categorical variables, each radiologist performed independent evaluations of the images; a kappa value greater than 0.75 was interpreted as indicating good agreement. Any discrepancies between the radiologists were resolved through consensus discussions. If an initial consensus was not reached, further deliberations were conducted until a final agreement was reached.

### 2.4 Pathological evaluation

All 25 patients diagnosed with ESC underwent radical esophagectomy with regional lymph node dissection. The diagnostic criteria for ESC were as follows (1) tumor originating within the esophagus; and (2) histopathological diagnosis of ESC or ESCC exhibiting sarcomatous differentiation. Pathological assessment included the evaluation of tumor invasion depth, thickness, and length. Additional pathological parameters examined included proportion of sarcomatoid component, lymphovascular invasion (LVI) status and perineural invasion (PNI) status. Tumor T and N stages were assigned according to the 8th edition of the American Joint Committee on Cancer (AJCC)/Union for International Cancer Control (UICC) TNM staging system.

### 2.4 Statistical analysis

Continuous variables that conform to a normal distribution were expressed as mean ± standard deviation (SD), whereas those that do not conform to a normal distribution were presented as median (interquartile range, IQR). The independent samples t-test was used to compare continuous variables that are normally distributed, while the Mann-Whitney U test was applied to continuous variables that are not normally distributed. Categorical variables were summarized as counts and percentages (%) and compared using the chi-square test or Fisher’s exact test. The ICC analysis was employed to evaluate interobserver agreement for quantitative analysis, with interpretations as follows: poor agreement for ICC ≤ 0.50; moderate agreement for 0.50< ICC ≤ 0.75; good agreement for 0.75 < ICC ≤ 0.90; and excellent agreement for ICC > 0.90. For qualitative analysis, Cohen’s kappa analysis was utilized, with kappa values interpreted as follows: fair agreement for 0.20-0.40; moderate agreement for 0.41-0.60; good agreement for 0.61–0.80; and excellent agreement for kappa values greater than 0.80.

The Bland-Altman analysis was performed to assess the agreement between CT measurements and pathological results. Scatter plots were generated to visually illustrate the measurement discrepancies between pTT and cTT, as well as between pTL and cTL. Additionally, Pearson or Spearman correlation analyses were employed to assess the relationship between CT measurements and pathological measurements, aiming to determine the extent of their association. All statistical analyses were performed using MedCalc version 4.30 (MedCalc Software, Mariakerke, Belgium).

## 3 Results

### 3.1 Clinical and pathological characteristics

Of the 25 patients with ESC, 19 were male and 6 were female. The patients’ ages ranged from 47 to 73 years, with a mean age of 65.48 ± 6.85 years and a median age of 68 years. All patients presented with varying degrees of dysphagia; additionally, retrosternal pain was reported in four cases. All patients underwent surgical resection, and postoperative pathological examination confirmed the diagnosis of ESC.

According to the macroscopic pathological classification, there was one case of the ulcerative type, seven cases of the elevated type, seven cases of the fungating type, and ten cases of the medullary type. Regarding macroscopic pathological classification, there was 1 case of ulcerative type, 7 cases of elevated type, 7 cases of fungating type, and 10 cases of medullary type. Tumor staging revealed 14 cases at T1 stage, 5 at T2 stage, 5 at T3 stage, and 1 at T4 stage. Lymph node metastasis was observed in 12 patients, including 5 cases classified as N1, 4 as N2, and 3 as N3 stage. The pTT ranged from 0.50 to 4.00 cm, with a mean of 1.92 ± 1.02 cm and a median of 1.50 cm; the pTL ranged from 2.00 to 7.00 cm, with a mean of 4.24 ± 1.50 cm and a median of 4.00 cm. Regarding the proportion of sarcomatoid components, the mass-forming type group exhibited a prevalence of 80.00% [IQR: 70%–90%]), whereas the wall-thickening type group had a prevalence of 50.00%[IQR: 50%-60%]. The difference between the two groups was statistically significant (P = 0.010).

With respect to main clinical symptoms, 21 patients presented with dysphagia only, 2 patients with retrosternal pain only, and 2 patients with both dysphagia and retrosternal pain. The detailed clinical and pathological characteristics of the patients are summarized in **Table 1**.

**Table 1:** Clinical and pathological characteristics of the 25 ESC patients.

Variables	Total	Mass-forming type (n=20)	Wall-thickening type (n=5)	P
Sex, n (%)				0.562^%^
male	19(76.00)	16(80.00)	3(60.00)	
female	6(24.00)	4(20.00)	2(40.00)	
Age, years	65.48 ± 5.85	64.40 ± 6.03	69.80 ± 1.47	0.069^&^
Main symptoms,n (%)				0.262^%^
dysphagia	21(84.00)	18(90.00)	3(60.00)	
retrosternal pain	2(8.00)	1(5.000)	1(20.00)	
both	2(8.00)	1(5.000)	1(20.00)	
T stage, n (%)				0.070^%^
1-2	19(76.00)	17(85.00)	2(40.00)	
3-4	6(24.00)	3(15.00)	3(60.00)	
N stage, n (%)				0.160^%^
0	13(52.00)	12(60.00)	1(20.00)	
1	5(20.00)	2(10.00)	3(60.00)	
2-3	7(28.00)	6(30.00)	1(20.00)	
LVI, n (%)				1.000^%^
negative	23(92.00)	18(90.00)	5(100.00)	
positive	2(8.00)	2(10.00)	0 (0)	
PNI, n (%)				0.038^%^
negative	20(80.00)	18(90.00)	2(40.00)	
positive	5(20.00)	2(10.00)	3(60.00)	
pTT, cm	1.92 ± 1.02	2.16 ± 1.00	0.98 ± 0.33	<0.001^@^
pTL, cm	4.24 ± 1.50	4.45 ± 1.54	3.40 ± 0.97	0.178^&^
Sarcomatoid component (%)	70.00[60.00,90.00]	80.00[70.00,90.00]	50.00[50.00,60.00]	0.010^*^

^&^t-test; ^@^Welch’s t-test; ^%^Fisher’s exact test; ^*^Mannwhitney-U.

### 3.2 CT features analysis

For the measurement and evaluation of CT features, Radiologist 1 and Radiologist 2 demonstrated good to excellent interobserver consistency. The ICC and kappa values for the CT features ranged from 0.818 to 0.969, indicating strong interobserver agreement. The detailed analysis results are as follows:

In the 25 patients with ESC, the cTT ranged from 0.60 to 4.10 cm, with a mean of 2.02 ± 0.90 cm and a median of 1.66 cm. The cTL ranged from 2.57 to 7.50 cm, with a mean of 4.41 ± 1.48 cm and a median of 4.16 cm. The cTV ranged from 1.56 to 41.49 cm³, with a median of 8.25 cm³ (IQR: 4.37–20.24). The CT_Tumor_ was 70.87 ± 19.94 Hu, while the CT_Normal_ was 39.51 ± 4.36 Hu. The ΔTN was 31.36 ± 17.87 Hu and the TNR was 1.81 (IQR: 1.47–2.02).

Among the 25 patients, 7 masses demonstrated necrosis; 12 showed homogeneous enhancement, while 13 exhibited heterogeneous enhancement. Clear tumor borders were observed in 15 cases, whereas 10 cases presented with unclear borders. Based on the CT characteristics of the masses, the 25 ESC cases were classified into two types: mass-forming type (n = 20) and wall-thickening type (n = 5).

The mass-forming type is defined as a nodular lesion growing along one side of the esophageal wall and protruding into the esophageal lumen, typically presenting as a well-defined mass. In contrast, the wall-thickening type is characterized by tumor growth along the esophageal wall, resulting in localized or circumferential thickening of the esophageal wall. Statistical analysis revealed that both pTT and cTT were significantly greater in the mass-forming type group than in the wall-thickening type. However, no statistically significant differences were observed between the two groups in terms of CT_Tumor_, TNR, or enhancement pattern. Detailed analysis of CT features in the patients is summarized in **Table 2**.

**Table 2:** The CT feature analysis of the 25 ESC patients.

Variables	Total	Mass-forming type (n=20)	Wall-thickening type (n=5)	P
CT_Normal_, mean ± SD (HU)	39.51 ± 4.36	39.81 ± 4.14	38.32 ± 4.98	0.516^&^
CT_Tumor_, mean ± SD (HU)	70.87 ± 19.94	71.99 ± 21.27	66.40 ± 12.40	0.594^&^
TNR	1.81[1.47,2.02]	1.92[1.47,2.10]	1.81[1.74,1.86]	0.621^*^
ΔTN	31.36 ± 17.87	32.18 ± 19.40	28.08 ± 8.83	0.663^&^
cTV, median [IQR]	8.25[4.37,20.24]	10.44[4.37,20.24]	8.20[6.11,10.76]	0.668^*^
cTL, mean ± SD (cm)	4.41 ± 1.48	4.59 ± 1.52	3.72 ± 1.07	0.260^&^
cTT, mean ± SD (cm)	2.02 ± 1.00	2.23 ± 0.87	1.18 ± 0.33	<0.001^@^
Necrosis, n (%)				0.597^%^
without	18(72.00)	15(75.00)	3(60.00)	
with	7(28.00)	5(25.00)	2(40.00)	
Enhancement pattern, n (%)				0.645^%^
homogeneous	12(48.00)	9(45.00)	3(60.00)	
heterogeneous	13(52.00)	11(55.00)	2(40.00)	
Margin pattern, n (%)				0.358^%^
clear	15(60.00)	13(65.00)	2(40.00)	
unclear	10(40.00)	7(35.00)	3(60.00)	

^&^t-test; ^*^Mann-Whitney U; ^@^Welch’s t-test; ^%^Fisher’s exact test.

**Figure 2:**
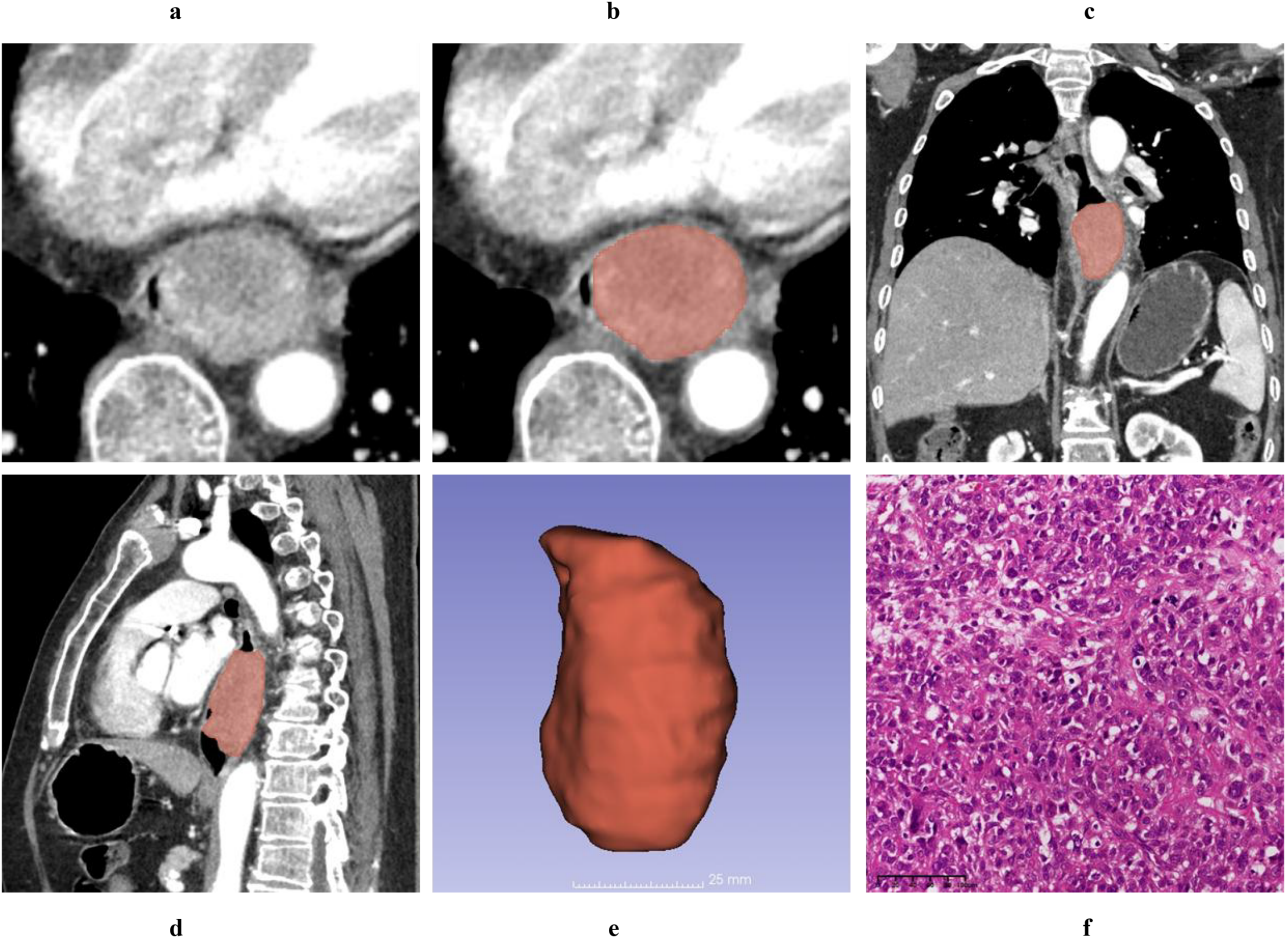
A 61-year-old male patient was diagnosed with a T1 stage mass-forming type ESC by postoperative pathology. Axial non-contrast computed tomography (CT) image **(A)** and contrast-enhanced CT (CECT) image **(B)** demonstrate a broad-based intraluminal mass in the distal esophagus, characterized by focal eccentricity, marked luminal narrowing, and heterogeneous enhancement. The tumor thickness measures 4.12 cm on the axial CECT image **(B)**. Multiplanar reconstruction (MPR) views in the coronal **(C)** and sagittal **(D)** planes clearly delineate the tumor’s size and extent, with the cTL measuring 6.32 cm on the sagittal CECT image **(D)**. Using 3D Slicer software, the tumor boundaries were meticulously delineated slice by slice to generate a three-dimensional region of interest (3D-ROI) **(E)**. Hematoxylin and eosin (HE, magnification ×20) staining of the tumor revealed spindle-shaped tumor cells, prominent atypical nuclei, pleomorphic nuclei, and giant nuclei **(F)**.

**Figure 3:**
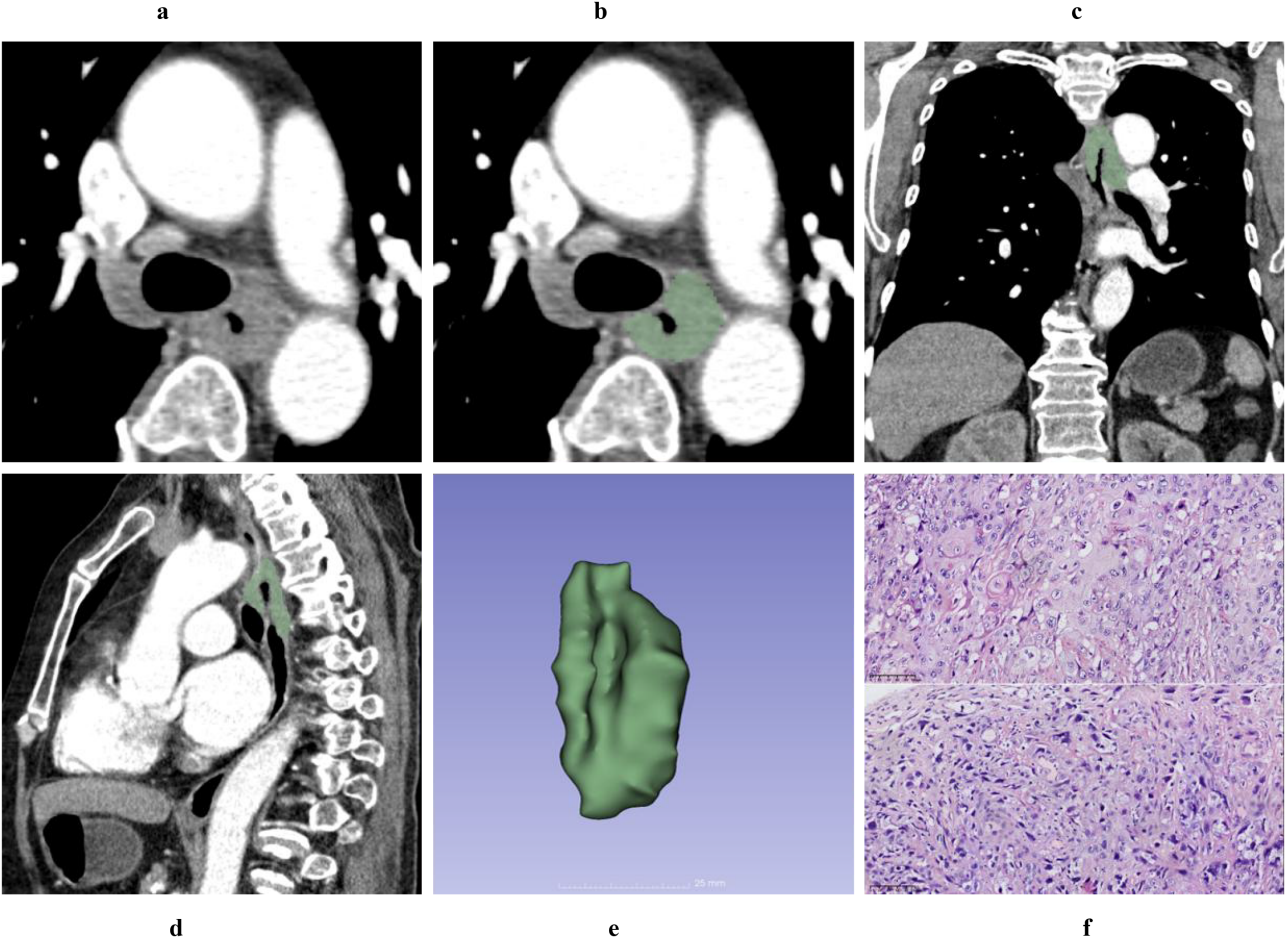
A 69-year-old female diagnosed with T3 stage, wall-thickening type ESC by postoperative pathology. Axial non-contrast computed tomography (CT) image **(A)** and contrast-enhanced CT (CECT) image **(B)** demonstrate marked circumferential irregular thickening of the middle esophageal wall, accompanied by significant central luminal narrowing and homogeneous enhancement. The tumor thickness measures 4.21 cm on the axial CECT image **(B)**. Multiplanar reconstruction (MPR) views in the coronal **(C)** and sagittal **(D)** planes clearly delineate the size and extent of the tumor, with the tumor length measuring 6.32 cm on the sagittal CECT image **(D)**. Using 3D Slicer software, the tumor boundaries were meticulously delineated slice by slice to generate a three-dimensional region of interest (3D-ROI) **(E)**. Hematoxylin-eosin (HE, magnification ×20) staining of the tumor revealed that many nests of squamous cell carcinoma were interspersed with sarcomatoid components, constituting approximately 50% of the tumor **(F)**.

Typical mass-forming type ESC appears as a large soft-tissue mass protruding into the esophageal lumen, exhibiting well-defined borders, regular shape, and either heterogeneous or homogeneous enhancement. The adjacent esophageal wall is slightly thickened, and the lumen is eccentrically narrowed to form a crescent shape (**Figure 2**). Typical wall-thickening type ESC manifests as focal or diffuse thickening of the esophageal wall with ill-defined boundaries between the lesion and normal esophagus tissue. It demonstrates either homogeneous or heterogeneous enhancement and is accompanied by varying degrees of central luminal narrowing (**Figure 3**).

### 3.3 The agreement and correlation analysis between CT features and pathological characteristics

**Figure 4:**
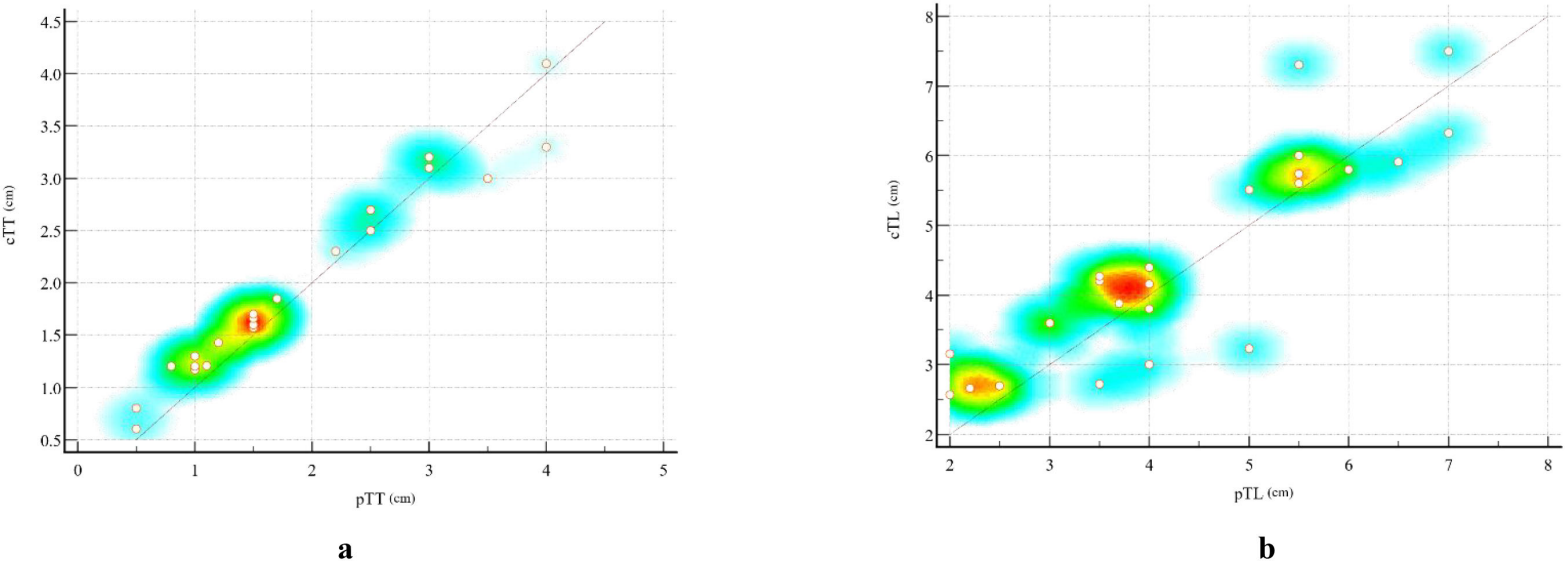
Scatter plots illustrating the correlation between CT-measured and pathological measurements of esophageal sarcomatoid carcinoma (ESC). **(A)** Scatter plot showing the relationship between cTT and pTT. **(B)** Scatter plot showing the relationship between cTL and pTL. Each dot represents one patient (n = 25). The diagonal line indicates the line of equality (y = x). The background color gradient from red to yellow to blue represents the local density of data points, with red indicating areas of highest concentration and blue corresponding to regions with sparse distribution. This color mapping visually highlights the clustering of cases with similar measurement values.

**Figure 5:**
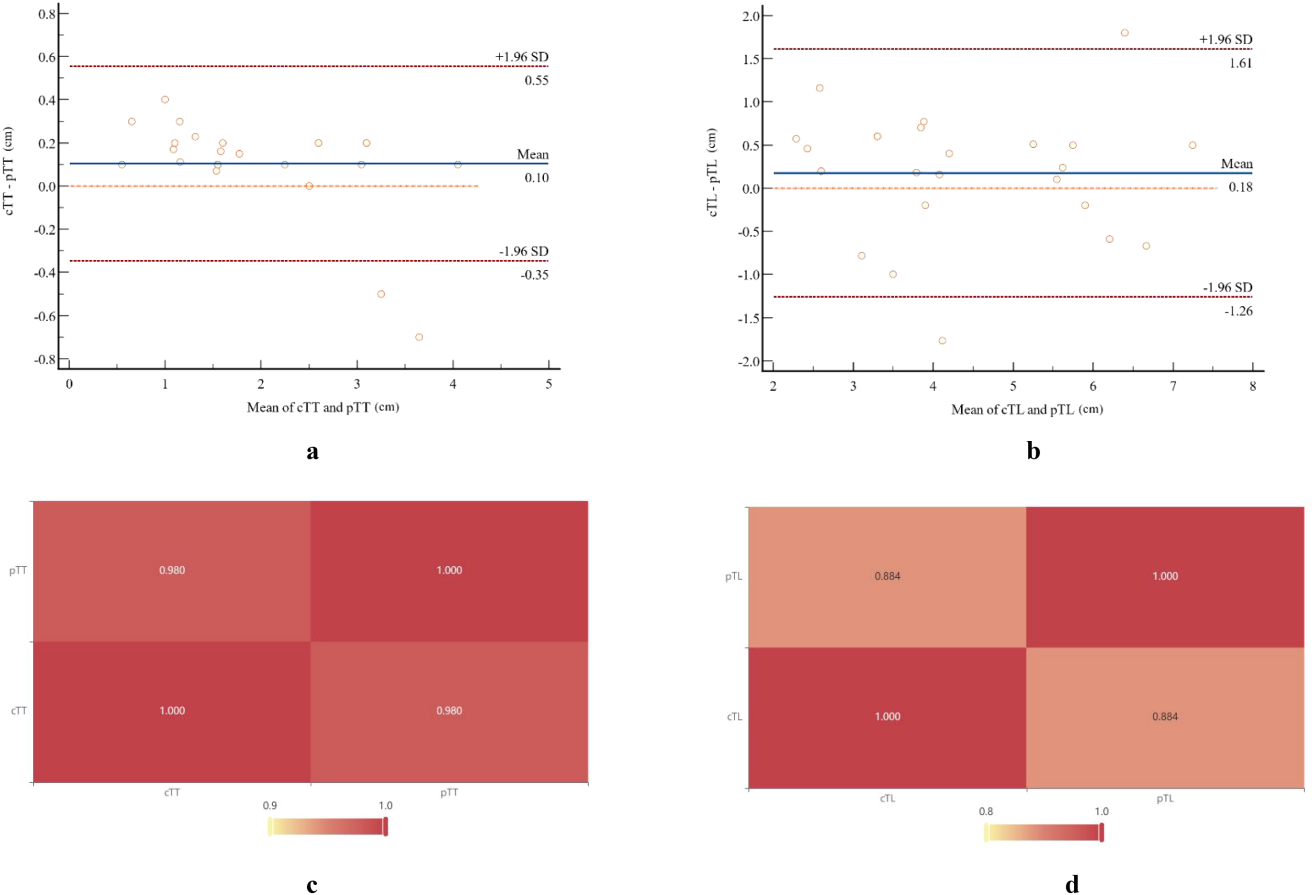
Bland-Altman plots and a heat map illustrate the comparison between pathological measurements and CT-measured tumor parameters. Bland-Altman analysis showed a mean difference of 0.10 cm between cTT **(A)** and pTT **(B)**, with 92.00% (23/25) of cases falling within the limits of agreement (mean ± 1.96×SD). The Pearson correlation coefficient between these two measurements was 0.980. Similarly, Bland-Altman analysis revealed a mean difference of 0.18 cm between cTL **(C)** and pTL **(D)**, with 92.00% (23/25) of cases within the limits of agreement. The Pearson correlation coefficient for these measurements was 0.884.

The specific values of cTL and pTL for the 25 ESC patients, together with their distributions and correlations, are illustrated in **Figure 4**. Bland-Altman plot analysis showed a mean difference of 0.10 cm between cTT and pTT, with 92.00% (23/25) of the cases falling within the limits of agreement (Mean ± 1.96×SD) (**Figure 5A**). The Pearson correlation coefficient between cTT and pTT was 0.980, indicating a strong positive correlation (**Figure 5C**). Similarly, Bland-Altman analysis of cTL and pTL revealed a mean difference of 0.18 cm, with 92.00% (23/25) of cases within the limits of agreement (Mean ± 1.96×SD) (**Figure 5B**). The Pearson correlation coefficient between cTL and pTL was 0.884, reflecting a strong positive correlation as well (**Figure 5D**). These results suggest that CECT provides relatively accurate quantitative measurements of tumor thickness and length in patients with ESC. Furthermore, Spearman’s rank correlation analysis was performed to assess the relationship between the proportion of sarcomatoid components and CT_Tumor_. This analysis revealed a weak positive correlation between the two variables (Spearman’s ρ = 0.103, P > 0.05; α = 0.05), suggesting no statistically significant association.

## 4 Discussion

SC is an uncommon tumor in the gastrointestinal tract, primarily arising in the esophagus, stomach, and gallbladder, and less frequently occurring in the large and small intestines (15). According to the 2002 edition of the World Health Organization’s Pathology and Genetics of Tumors of the Digestive System, ESC is defined as a distinct variant of esophageal epithelial malignancy, typically characterized by the coexistence of squamous cell carcinoma components and a heterogeneous sarcomatous spindle cell component. Consequently, ESC is classified as a spindle cell carcinoma (16). Epidemiologically, ESC is more commonly observed in males, predominantly affecting individuals aged 60 to 70 years. Approximately 60% of ESC cases originate in the middle esophagus, nearly one-third located in the lower esophagus, and fewer than 10% occurring in the upper esophagus (7, 17).

Histopathologically, SC may exhibit either a biphasic or monophasic pattern. The typical biphasic pattern is characterized by a mixture of epithelial-like and mesenchymal-like cells, whereas monophasic tumors predominantly consist of the mesenchymal-like component with minimal or absent epithelioid areas. Immunohistochemical investigations have revealed that sarcomatoid cells frequently exhibit immunoreactivity to cytokeratins, substantiating the hypothesis that these cells originate from epithelial tissue (8, 18). Several studies have emphasized that the sarcomatoid component should constitute more than 50% of the tumor mass to meet diagnostic criteria (10, 11, 16, 19). Due to their frequent polypoid growth projecting into the esophageal lumen, these tumors tend to cause obstructive symptoms at an earlier stage, resulting in a relatively shorter disease course. ESC typically exhibits this polypoid growth pattern, which contributes to the earlier onset of obstructive symptoms and is associated with a comparatively favorable prognosis. On gross pathological examination, ESC usually presents as polypoid masses that can reach up to 15 cm in diameter. These masses are generally pedunculated but may occasionally be sessile, often demonstrating extensive adhesion to the esophageal mucosa (7).

Multi-slice CT provides advanced post-processing capabilities, allowing detailed visualization of tumor size, shape, location, and the extent of adjacent esophageal wall thickening. This facilitates the assessment of extramural invasion and lymph node metastasis. However, to date, no comprehensive, systematic, and detailed scientific description of the CT features specific to ESC has been published, and only a limited number of case reports are available. Zhao et al. (17) reported a case involving a 63-year-old man with ESC, presenting as a regularly slightly lobulated mass growing intracavitarily in the lower esophagus. On non-contrast CT images, the mass exhibited unclear boundary and uneven edges. CECT showed uniform, persistent enhancement during both arterial and venous phases, with more prominent central enhancement relative to the periphery. This pattern of enhancement was attributed to the abundant sarcomatoid component and rich intratumoral blood supply. SK et al. (20) reported reported a case of a 56-year-old male with ESC presenting with fever. Chest CECT revealed a 10-cm circumferential mass in the mid-thoracic esophagus without evidence of surrounding abscess; however, other detailed CT features were not described. Phelps et al. (21) reported a case of a 92-year-old female with ESC presenting with acute dysphagia. Sagittal and coronal contrast-enhanced CT images revealed a large, rounded mass containing gas and fluid, resulting in esophageal obstruction. Notably, axial CT images and additional analyses of CT features were not provided. Chen et al. (22) described a 51-year-old man with histopathologically and immunohistochemically confirmed ESC. Non-contrast chest CT image revealed a rounded mass measuring 95 × 45 × 28 mm in the mid-lower esophagus, causing significant luminal narrowing. Axial and coronal CECT images showed moderate, homogeneous tumor enhancement; however, sagittal image and additional CT details were not provided. Overall, these reports are limited by incomplete or non-systematic descriptions of the CT features associated with ESC. Therefore, our study aims to analyze the CT features of ESC from a novel and comprehensive perspective.

Our results showed that ESC primarily manifested as mass-forming type lesions, accounting for 82.61% (20/25) of cases. This observation is consistent with previous case reports and literature reviews. When comparing tumor measurements obtained by CT with postoperative pathological findings, we observed strong correlations: the Pearson correlation coefficients were 0.980 for tumor thickness and 0.884 for tumor length. Bland-Altman analysis showed mean differences of 0.10 cm in thickness and 0.18 cm in length between CT measurements and pathological results, with 92.00% of cases for both parameters falling within the limits of agreement (mean ± 1.96 × SD). These findings indicate that CT can accurately quantify the morphological dimensions of ESC tumors. However, traditional two-dimensional measurements offer limited intuitive insight into tumor morphology. To address this, we employed 3D Slicer software to measure tumor volume and generate comprehensive 3D reconstructions, thereby facilitating a more holistic visualization of the entire tumor.

Regarding primary gastric SC, Liu et al. (23) classified tumors into two subtypes based on CT features: the wall-thickening type and the tumor mass-forming type. For SC originating in the small bowel, several case reports (24, 25) indicated that most tumors present as mass-forming type lesions with irregular or rounded shapes on CT. These masses typically show an intraluminal growth pattern, resulting in luminal narrowing and proximal small bowel dilatation, and demonstrate poor, heterogeneous enhancement on CECT. Conversely, two other case reports (15, 26) suggested that small bowel SC may also present as localized circumferential or diffuse irregular thickening of the bowel wall accompanied by luminal narrowing. Similarly, based on CT features of 25 ESC patients, we classified tumors into mass-forming and wall-thickening types. The majority (20/25) exhibited a mass-forming type morphology, characterized by pronounced eccentric luminal narrowing, while only 5 cases displayed the wall-thickening type. Pathologically, these five wall-thickening type ESCs predominantly showed a medullary pattern and appeared as peripheral or localized thickening on CT images. Quantitatively, the pTT and cTT of the mass-forming type tumors were significantly greater than those of the wall-thickening type. However, other CT features showed no significant differences between the two subtypes.

This present study reveals that cases classified as stages T1–2 comprised 76% (19 out of 25), with 48% (12 out of 25) of ESC patients presenting lymph node metastasis. Notably, the lymph node metastasis rate for the wall thickening subtype was as high as 80% (4 out of 5). These findings suggest that ESC typically does not extensively invade the esophageal wall; however, even at early T stages, there can be occurrences of highly aggressive lymph node metastasis. This conclusion aligns with findings from previous research (27). Mass-forming type ESC typically presents with well-defined boundaries, a regular shape with slight lobulations, and uniform density on CT images, whereas necrotic cystic degeneration is uncommon. These CT features markedly differ from those of ESCC, which is typically characterized by ring-like wall hyperplasia and thickening, with necrosis frequently observed in larger tumors. Previous case reports on ESC have described its enhancement characteristics; however, there remains a lack of quantitative analysis providing specific numerical data (17, 20, 28). In the present study, it was observed that the proportion of sarcomatoid carcinoma components was significantly higher in the mass-forming type ESC compared to the wall-thickening type (P < 0.05). This suggests that ESC with a greater sarcomatoid component tends to present as mass-forming lesions, whereas those with fewer sarcomatoid components more commonly display CT features similar to ESCC.

In the present study, we found that the CT_Tumor_ of mass-forming ESC were slightly higher than those of wall-thickening ESC; however, this difference was not statistically significant (P > 0.05). To some extent, ESC with a higher proportion of sarcomatoid components showed increased CT values. Nonetheless, Spearman correlation analysis demonstrated only a weak positive correlation between CT_Tumor_ and the proportion of sarcomatoid components. Furthermore, the relationship between sarcomatoid and epithelial components concerning enhancement patterns remains unclear and warrants further investigation. Notably, the CT_Tumor_ of mass-forming type ESC closely resembled that observed in ESCC cases with LVI, whereas the CT_Tumor_ of wall-thickening type ESC was comparable to that of ESCC without LVI (29). These findings highlight the complexity of CT imaging features in ESC and underscore the need for additional studies to clarify the correlation between histopathological components and imaging characteristics. Nevertheless, further comparative studies examining the degree and pattern of contrast enhancement between ESC and ESCC are necessary. This constitutes a promising direction for our ongoing research.

## 5 Conclusions

In conclusion, ESC is a rare tumor characterized by distinctive CT features. The majority of cases present as the mass-forming type, whereas a minority exhibit the wall-thickening type. The CT features of ESC identified in our study may offer valuable insights for future investigations. However, an accurate diagnosis of ESC depends on an integrated approach that combines clinical presentation, multimodal imaging findings, and histopathological analysis.

## Data Availability

The original contributions presented in the study are included in the article/supplementary material. Further inquiries can be directed to the corresponding authors.
